# The Role of Actin Cytoskeleton in Dendritic Spines in the Maintenance of Long-Term Memory

**DOI:** 10.3389/fnmol.2018.00143

**Published:** 2018-05-01

**Authors:** Sreetama Basu, Raphael Lamprecht

**Affiliations:** Sagol Departmant of Neurobiology, Faculty of Natural Sciences, The Integrated Brain and Behavior Research Center, University of Haifa, Haifa, Israel

**Keywords:** actin cytoskeleton, dendritic spines, long term memory, memory maintenance, structural plasticity, neuronal morphology

## Abstract

Evidence indicates that long-term memory formation involves alterations in synaptic efficacy produced by modifications in neural transmission and morphology. However, it is not clear how such alterations induced by learning, that encode memory, are maintained over long period of time to preserve long-term memory. This is especially intriguing as the half-life of most of the proteins that underlie such changes is usually in the range of hours to days and these proteins may change their location over time. In this review we describe studies that indicate the involvement of dendritic spines in memory formation and its maintenance. These studies show that learning leads to changes in the number and morphology of spines. Disruption in spines morphology or manipulations that lead to alteration in their number after consolidation are associated with impairment in memory maintenance. We further ask how changes in dendritic spines morphology, induced by learning and reputed to encode memory, are maintained to preserve long-term memory. We propose a mechanism, based on studies described in the review, whereby the actin cytoskeleton and its regulatory proteins involved in the initial alteration in spine morphology induced by learning are also essential for spine structural stabilization that maintains long-term memory. In this model glutamate receptors and other synaptic receptors activation during learning leads to the creation of new actin cytoskeletal scaffold leading to changes in spines morphology and memory formation. This new actin cytoskeletal scaffold is preserved beyond actin and its regulatory proteins turnover and dynamics by active stabilization of the level and activity of actin regulatory proteins within these memory spines.

Evidence suggests that long-term memory is formed by enduring alterations in synaptic efficacy and connectivity between neurons (Konorski, 1948; Hebb, 1949; Dudai, 1989; Bliss and Collingridge, 1993; Martin et al., 2000; Tsien, 2000; Kandel, 2001; Lamprecht and LeDoux, 2004; Caroni et al., 2012; Bailey et al., 2015). However, it is not clear how such changes induced by learning that encode memory are maintained over long period of time to preserve long-term memory especially since the half-life of the proteins that underlie such changes is relatively short (Hanus and Schuman, 2013; Alvarez-Castelao and Schuman, 2015) and these proteins may change their location over time. In this review we will explore the roles of dendritic spines in long-term memory maintenance and examine the possibility that morphological changes that are induced by learning and that are hypothesized to encode memory are maintained to preserve long-term memory without significant decay. We will further describe how the actin cytoskeleton may be involved in preserving the morphology of dendritic spines after learning to maintain long-term memory.

## Spine morphology affects neuronal function

Dendritic spines receive excitatory synaptic inputs and confine local synaptic signaling and the diffusion of postsynaptic molecules (Nimchinsky et al., 2002; Lamprecht and LeDoux, 2004; Newpher and Ehlers, 2009; Nishiyama and Yasuda, 2015). Alterations in spine morphology may be involved in neuronal functions that subserve memory formation. For example, it was revealed that spines with large postsynaptic densities (PSDs) tend to have a higher level of α-amino-3-hydroxy-5-methyl-4-isoxazolepropionic acid receptors (AMPARs) than spines with smaller PSDs (e.g., Takumi et al., 1999). Since the area of PSDs is correlated with that of the dimensions of the spine head (Harris and Stevens, 1989), it is implied that spines with larger head express more glutamate receptors than spines with smaller head. In addition, a study found a correlation between the amplitudes of currents in the spine and the spine head volume showing that the distribution of functional AMPARs is approximately proportional to the spine head volume (Noguchi et al., 2011). Thus, synaptic efficacy mediated by AMPA receptors is correlated with spine head volume from silent synapses in small spines to highly responsive larger spines. AMPA receptors trafficking into the synapse is involved in memory formation. For example, fear conditioning drives glutamate receptor 1 (GluA1)-containing AMPARs into synapses in lateral amygdala (LA) neurons (Rumpel et al., 2005; Yeh et al., 2006; Nedelescu et al., 2010; Ota et al., 2010). Moreover, fear memory is impaired if GluA1-AMPAR insertion is blocked (Rumpel et al., 2005).

The geometry of the spine neck may also affect synaptic efficacy. Spine neck plasticity appears to mainly affect local voltage amplification in spines and biochemical compartmentalization, such as of Ca^2+^, within the spine head (Noguchi et al., 2005) that may affect signal transduction and bidirectional diffusion of material from dendrite to spines (Bloodgood and Sabatini, 2005; Gray et al., 2006; Santamaria et al., 2006). Spines with longer thinner spine necks confine more molecules. Thus, changes in spine neck may affect synaptic efficacy and also neuronal function (Araya et al., 2006, 2014). For example, spines with long neck have small somatic voltage contributions. Synaptic stimulation paired with postsynaptic activity can lead to shortening of spines necks and to change in the input/output gain of pyramidal neurons and to increase in synaptic efficacy (Araya et al., 2014).

## Learning leads to spines morphogenesis

It has been shown that changes in dendritic spines morphology and number are associated with memory formation (Lamprecht and LeDoux, 2004; Bailey et al., 2015). For example, contextual fear conditioning leads to an increase in the density of dendritic spines in hippocampal CA1 and the anterior cingulate cortex (Restivo et al., 2009; Vetere et al., 2011). Auditory fear conditioning increases the rate of spines elimination in layer-V pyramidal neurons in the mouse frontal association cortex whereas fear extinction induces spines formation in this brain region (Lai et al., 2012). Fear conditioning leads to an increase in postsynaptic density (PSD) area in smooth endoplasmic reticulum (sER)-free spines and to decrease in spines head volume in LA (Ostroff et al., 2010). Intense training with high footshock during inhibitory avoidance, that induced higher resistance to extinction and thus suggests an enhanced learning, led to an increase in mushroom shaped spines along with a decrease in thin spines in the dorsomedial striatum (Bello-Medina et al., 2016). Auditory fear conditioning leads to increase in pathway-specific formation of LA axons boutons in auditory cortex (ACx), dendritic spines of pyramidal cells in layer 5 of ACx, and putative LA–ACx synaptic pairs (Yang et al., 2016).

## Spines stability and long-term memory

A key question that arises from the above observations is whether spines formation and morphogenesis induced by learning are stable for a long period of time to maintain long-term memory. Evidence indicates that this may be the case. First, there are ample observations showing that spines are stable for days to years. For example, the structure of dendritic spines is stable for days in cultured hippocampal slices (De Roo et al., 2008) and for years in the cortex *in vivo* (Grutzendler et al., 2002; Trachtenberg et al., 2002; Zuo et al., 2005). Second, spine stability is associated with long-term memory persistence. For example, a fraction of newly formed spines persist over weeks and the amount of stable spines correlates with performance after learning (Yang et al., 2009). New dendritic spines are grown following training for a forelimb reaching task and are preferentially stabilized by subsequent training sessions (Xu et al., 2009). Acquired motor task is disrupted by post learning optical activation of Rac1 GTPase and shrinkage of the learning-potentiated spines a day after training indicating that preserving the spines morphology is necessary for memory maintenance and that their shrinkage leads to memory erasure (Hayashi-Takagi et al., 2015). Interfering with actin cytoskeleton polymerization in basolateral amygdala complex (BLC) during the maintenance phase of conditioned place preference (CPP) memory led to the impairment in maintenance of CPP memory and to decrease in spines density in BLC suggesting that dendritic spines persistence supports the maintenance of the memory trace (Young et al., 2014).

The above observations show that spines are formed by learning and last for days to weeks and potentially more after behavioral training and that disruption in spines morphology after memory consolidation is associated with impairment in memory maintenance suggesting that spines persistence is essential for memory maintenance. However, it is not clear how these spines are stabilized in the face of the short life and dynamics of the molecules that build them. Below we suggest that the actin cytoskeleton which is intimately involved in spine formation and morphogenesis also stabilizes its structure under certain conditions, a stabilization that is necessary for maintaining long-term memory.

## Actin cytoskeleton is involved in spine morphogenesis and memory formation

### Actin and spine morphology

Actin cytoskeleton is involved in the morphogenesis of dendritic spines. Mature spines contain a mixture of branched and linear actin filaments at their base, neck, and head. The spine neck contains both linear and branched filaments whereas branched actin filament network is a dominant feature of the spine head (Korobova and Svitkina, 2010). The actin cytoskeleton is intimately involved in the formation and elimination, stability, motility, and morphology of dendritic spines (Halpain et al., 1998; Matus, 2000; Schubert and Dotti, 2007; Honkura et al., 2008; Hotulainen and Hoogenraad, 2010; Chazeau et al., 2014). The shape and dynamics of mature spines are regulated by two distinct pools of actin filaments (Honkura et al., 2008). The stable pool of F-actin has a turnover rate of minutes and is mainly found at the base of the spine head whereas the dynamic pool has a turnover rate of seconds. It is suggested that the volume of spines is maintained actively and continuously by an exact balance between the pressure generated by the surrounding tissue and the expansive force created by the dynamic F-actin pool. Changes in spine structure depend on actin polymerization. For example, spine head enlargement by glutamate stimulation is dependent on actin polymerization (Matsuzaki et al., 2004).

In addition to stabilization of spine head morphology actin may be involved also in spine neck stabilization. A biophysical model suggests that constriction of the spine neck assists in the stabilization of spines, thus pointing to a role in stabilization and maintenance of ring-like F-actin structures that are consistently found in spine neck (Miermans et al., 2017).

Actin cytoskeleton polymerization, depolymerization and branching leading to changes in spine morphology are closely controlled by small GTPases Rac1, Cdc42 and Rho GTPases and their downstream effectors such as Arp2/3 and formins (e.g., Luo, 2000; Woolfrey and Srivastava, 2016). These actin regulatory proteins are functionally linked with synaptic receptors, such as glutamate receptors, Eph receptors, and adhesion molecules (e.g., cadherin), that participate in spine morphogenesis and memory formation (e.g., Woolfrey and Srivastava, 2016). In addition, actin filaments dynamics may be also coupled with microtubules dynamics for temporal and local regulation of dendritic spines (Shirao and González-Billault, 2013).

### Actin and memory

It has been shown that actin cytoskeleton is essential for memory formation. Interfering with proper actin cytoskeleton polymerization impairs the formation of long-term memory (e.g., Mantzur et al., 2009; Rehberg et al., 2010; Gavin et al., 2011). Moreover, regulation of actin polymerization is important for spine morphology and memory formation. For example, deletion of the actin filament depolymerizing protein n-cofilin or its regulator LIM kinase (LIMK-1) leads to alterations in spines morphology, synaptic plasticity and learning and memory (Meng et al., 2002; Rust et al., 2010). In addition, interfering with cofilin function impaired spines shrinkage induced by LTD and memory extinction (Zhou et al., 2004; Wang et al., 2013). In addition, actin-regulatory proteins that control actin filaments network and affect spine morphology are also involved in memory formation. For example, the WAVE isoforms (WAVE-1, WAVE-2, and WAVE-3) allow the assembly of multiprotein complexes that include regulatory proteins that affect actin structure and branching (e.g., Arp2/3) (Pollard, 2007; Takenawa and Suetsugu, 2007; Pollitt and Insall, 2009). This Wave Regulatory Complex (WRC) is functionally linked to synaptic receptors to affect actin cytoskeleton and spine morphology. For example, BDNF signaling may activate Rac1, that in turn leads to relocation of CYFIP1 (cytoplasmic FMRP-interacting protein 1) to affect the WRC, actin cytoskeleton and spine morphology (De Rubeis et al., 2013). Loss of WAVE-1 reduces spines density and leads to impairment in Morris water maze memory retention (Soderling et al., 2007). Arp2/3 is concentrated in spines and is needed for spine head growth and for activity-dependent spine enlargement (Kim et al., 2006, 2013; Rácz and Weinberg, 2008; Wegner et al., 2008; Hotulainen et al., 2009). Deletion of ArpC3, an essential Arp2/3 subunit, leads to defects in actin turnover in spine and spine formation and morphology (Kim et al., 2013). ArpC3f/f:CamKllα-Cre mice are impaired in Y-maze (working memory) and novel object recognition (episodic memory) tests. Inhibition of Arp2/3, in LA during auditory fear conditioning impaired the formation of long-term, but not short-term, fear memory (Basu et al., 2016).

Thus, actin cytoskeleton and its regulatory proteins are involved in spine morphogenesis and memory formation. However, for memory to persist these structural changes need to be maintained over long-period of time. Can actin cytoskeleton maintain long-lasting changes in spine morphogenesis observed after learning?

## Evidence for a role for actin cytoskeleton and its regulatory proteins in maintaining spine morphology and memory

There are several observations that show that actin and its regulatory proteins are involved in maintaining spines morphology and the persistence of long–term memory. The maintenances of long-term conditioned place preference (CPP) memory, formed through association with methamphetamine (METH), is impaired by infusion of Latrunculin A (LatA), into basolateral amygdala complex (BLC) 2 days after training (Young et al., 2014) (LatA prevents the incorporation of G-actin into dynamic F-actin, Morton et al., 2000). Inhibition of non-muscle myosin II also impaired CPP memory maintenance. The investigators further revealed that spines density in BLC increased with CPP training and that LatA infusion into BLC 2 days following training reduced spines density in CPP-paired animals, with no effect on spines in control animals. Thus, the study implies that maintenance of memories is supported by a constitutive cycling of filament actin that maintains spine stability.

Actin regulatory proteins are also involved in maintaining long-term memory. Activation of Rac1, a GTPase that affects actin regulatory proteins, in activated synapses in motor cortex leads to spine shrinkage (Hayashi-Takagi et al., 2015). Activation of Rac1 a day after training also impaired motor task memory. Since activation of Rac1 leads to disruption of actin cytoskeleton and spine shrinkage (Hayashi-Takagi et al., 2015) the study indicates that the integrity of spines structure is important for maintaining motor task memory. In this context it is worth noting that loss of Rac1 leads to increase in mean PSD length and mean spine head area and impairment in working/episodic-like memory in the delayed matching-to-place (DMP) task (Haditsch et al., 2009). Thus, alteration in Rac1 activity leads to abnormal spine morphology and affects memory formation and maintenance.

Rac1 is also involved in forgetting. Inhibition of Rac1 activity in hippocampal neurons form extended object recognition memory and impairs the forgetting of contextual fear memory (Jiang et al., 2016; Liu et al., 2016). Rac1 activation on the other hand accelerated memory decay within 24 h. Moreover, expression of active Rac1 produced more lamellipodia-like synapses with a large spine head and overexpression of Rac1-DN led to a reduced spine density, with more long and thin filopodia-like spines. Activation of Rac1 in LA impaired long- but not short-term memory formation (Das et al., 2017). In Drosophila, Rac1 also mediates forgetting (Shuai et al., 2010; Dong et al., 2016).

Cdc42 is also involved in synaptic and structural plasticity of spines and in memory formation (Kim et al., 2014; Hedrick et al., 2016). Cdc42 cKO affects spine morphology, synaptic plasticity, and remote memory in mice (Kim et al., 2014). Cdc42 is also implicated in forgetting. Single-session training of Drosophila leads to anesthesia-resistant memory (ARM) formation and Cdc42 activation. Repeated learning extends ARM by inhibition of Cdc42-mediated forgetting. Inhibition of Cdc42 prolongs ARM retention and increased Cdc42 activity abolishes repetition-induced ARM extension (Zhang et al., 2016).

Forgetting is also regulated by Arp2/3 complex in C. elegans (Hadziselimovic et al., 2014). Upregulation of the Arp2/3 complex in AVA interneuron prevents forgetting. In contrast, downregulation of the Arp2/3 complex accelerates forgetting. Interestingly, it was shown that ArpC3 is needed for maintaining normal spine morphology in mice as ArpC3 deletion has no effect on spines morphology 1–2 weeks after ArpC3 knock down but at the 4 and 8 weeks time points the fraction of mushroom type spines decreased while filopodia-like spines increased in dendrites from ArpC3 KO compared to control (Kim et al., 2013).

Profilin is an actin regulatory protein that can mediate stabilization of spine morphology (Ackermann and Matus, 2003; Michaelsen et al., 2010; Michaelsen-Preusse et al., 2016). Profilin is translocated into dendritic spines after various stimulation such as stimuli leading to LTP or LTD and NMDA receptors stimulation (Ackermann and Matus, 2003; Michaelsen et al., 2010; Bosch et al., 2014; Michaelsen-Preusse et al., 2016). Profilin translocation into spines starts minutes after stimulation and lasted for many hours leading to suppression of actin dynamics and stabilization of spine morphology. Profilin–G-actin complex binds to VASP through its poly-proline segment (G(GP_5_)_3_) (Reinhard et al., 1995; Ferron et al., 2007) and such binding is needed for glutamate-induced translocation of profilin into spines and for consolidation and stabilization of spine morphology (Ackermann and Matus, 2003). It has been shown that fear conditioning leads to the translocation of profilin into dendritic spines in LA (Lamprecht et al., 2006) and that these profilin-containing spines in LA are larger than spines that do not contain profilin. Microinjection of G(GP_5_)_3_, that binds profilin and thus competes with its binding to VASP, but not the control peptide G(GA_5_)_3_, impaired the formation of long- but not short-term fear memory in LA (Basu et al., 2016). These results indicate that VASP-profilin binding in LA is essential for the formation of long-term fear memory. Moreover, it suggests that profilin translocation into spines, that leads to suppression of actin dynamics and stabilization of spine structure (Ackermann and Matus, 2003), is essential for the formation of long-term fear memory in LA.

The above observations indicate that the actin cytoskeleton and its regulatory proteins are involved in spine stabilization and in the maintenance of long-term memory. However, it is not clear how actin preserves spine stability that may mediate the maintenance of long-term memory. This is especially puzzling in light of the relatively short half-life of actin and its regulatory proteins and in the fast dynamic of the actin cytoskeleton and associated proteins network that support spine morphology.

## A model for actin cytoskeleton maintenance of spine structure and long-term memory

The above observations indicate that spines morphogenesis followed by their stabilization are involved in long-term memory formation and maintenances, respectively. Moreover, the actin cytoskeleton and its regulatory proteins are involved in spine morphogenesis and stabilization and in memory consolidation and maintenances. However, these observations beg the question: How spine structure stability involved in memory maintenance last beyond actin and its regulatory proteins turnover and dynamics to preserve enduring memories? Below are observations that collectively form a model to describe the function of actin cytoskeleton in spine stabilization and memory endurance.

The model includes two aspects that interact with each other- the spontaneous activation of glutamate receptor during the maintenance phase of memory to reduce actin dynamic and to tag the memory trace spines for delivery of proteins and mRNAs into spines and the maintenance of the actin network in spines in a steady state structure to preserve spines morphology.

### Spontaneous glutamate activity maintains actin structure, spine morphology and memory

It has been shown that actin dynamics in spines and actin-based protrusive activity from the spine head are potently inhibited by activation of either AMPA or NMDA receptors (Fischer et al., 2000). This blockade of motility causes spines to round up and to be more stable and regular. The authors further show results suggesting that low-voltage-activated Ca^2+^ channels mediate the inhibitory effects of AMPARs on actin dynamics in spine. The effect on reduction of actin dynamics could be mediated by Ca^2+^-responsive actin binding proteins. The authors suggest two distinct types of morphological plasticity in spines, the first leading to formation of new spines by stimulation such as LTP operating through NMDA receptors, and a second where AMPAR activation at established synapses stabilizes spine morphology. The differential involvement of glutamate in various stages of synapse formation may be related to the different conditions appropriate for spine formation and morphogenesis and for those required for spines maintenance. For example, newly formed synapses may exhibit only NMDA receptor-mediated currents followed by insertion of AMPA receptors (Liao et al., 1999; Petralia et al., 1999). As mentioned above learning leads to the insertion of AMPA receptors into synapses (e.g., Rumpel et al., 2005). Maintenance of established spine structure is suggested to require continual activation of AMPA receptors involving miniature synaptic events resulting from spontaneous vesicle fusion to prevent spine loss in the absence of action potentials (McKinney et al., 1999). Thus, miniature synaptic events at specific spines where responses to glutamate is enhanced during learning (e.g., by insertion of AMPA receptors) may lead to the maintenance of dendritic spines and their morphology needed for the persistence of long-term memory. Indeed, increase in miniature synaptic events is detected following learning during memory maintenance period (e.g., Ghosh et al., 2015). Thus, it could be that larger spines that contain more AMPA receptors are more sensitive to release of glutamate, and thus more stable, than smaller spines that are less sensitive and more dynamic. Indeed, larger spines are resistant to LTP and suggested to form the physical trace of long-term memory (Matsuzaki et al., 2004). Thus, the aforementioned observations suggest that glutamate receptors and calcium channels activation during learning leads to changes in actin structure and neuronal morphogenesis in specific activated spines. Subsequently, AMPA receptors activation by spontaneous release of glutamate in these synapses is involved in suppressing actin dynamic and preserving the new actin structure.

Activation of glutamate receptors can also lead to recruitment of actin capping proteins, known to stabilize F-actin, into spine head and can serve to stabilize spine morphology. Selective activation of synaptic glutamate receptors can lead to translocation of the actin filament barbed-end capping proteins Eps8, that stabilizes F-actin (Disanza et al., 2004), to the spine head (Menna et al., 2013). Eps8 is needed for proper spine morphology (Menna et al., 2013) and mice lacking Eps8 exhibit immature spines. These Eps8 KO mice are also impaired in passive avoidance long-term memory (Menna et al., 2013).

Reduction in actin dynamics induced by glutamate can support the stabilization of spines but it does not solve the problem of how actin cytoskeleton structure in spine, that mediates spine morphology, is preserved for long time despite actin protein turnover and dynamics. A self-perpetuating mechanism that maintains the structure of actin in spines is required to preserve spine morphology after learning.

### Learning leads to the formation of new scaffold of actin cytoskeletal structure that is preserved to maintain spine structure and long-term memory

As described above the structure of dendritic spines may be stable after learning for months or years. However, the actin filaments that support spines structure turn over in minutes to hours. Over 80% of F-actin in spines turns over every minute (e.g., Star et al., 2002). How therefore does the spine structure remain stable in light of the rapid turnover of actin cytoskeleton that supports its structure?

**Figure 1:**
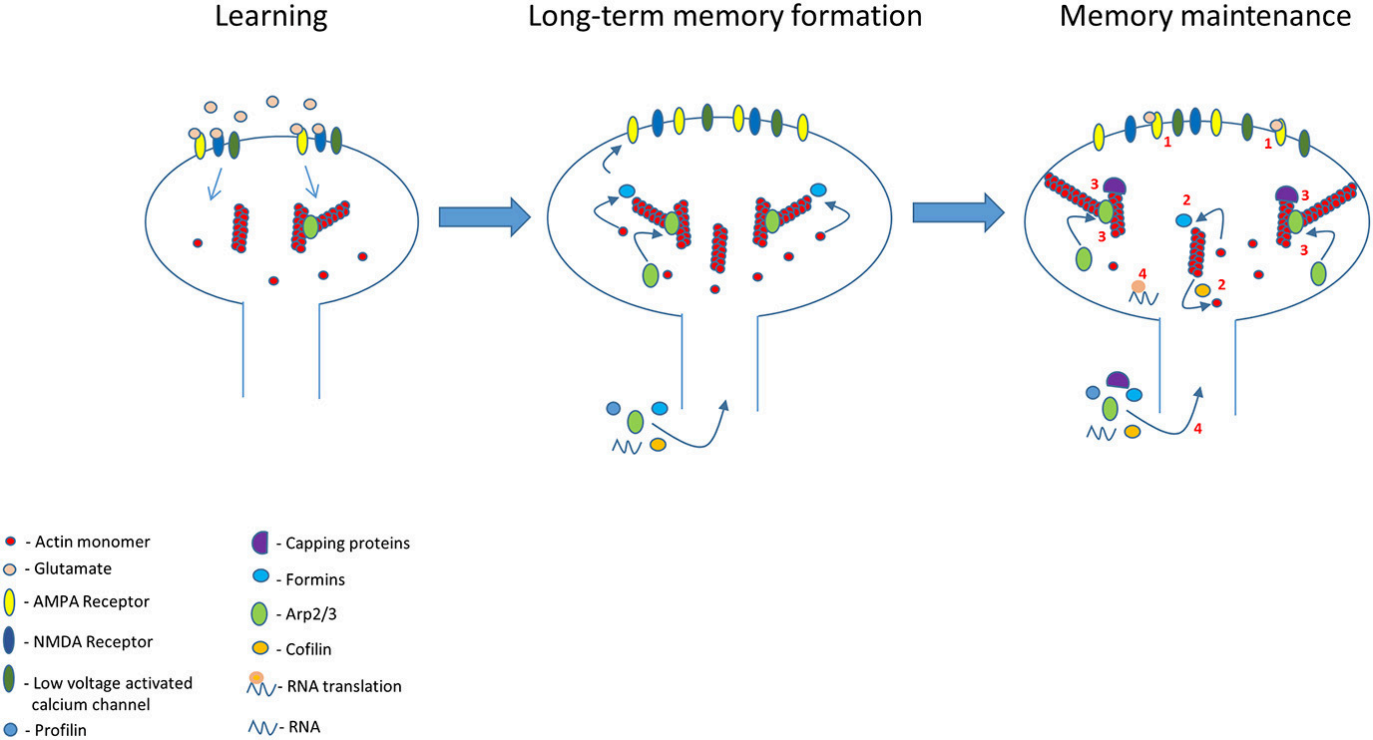
Glutamate receptors and other synaptic receptors (e.g., Eph receptor) activation during learning leads to build up of new actin cytoskeletal scaffold leading to changes in spines morphology and memory formation. This new actin cytoskeletal scaffold is preserved in the memory spines to maintain spine morphology and long-term memory. This actin cytoskeleton structure lasts beyond actin and its regulatory proteins turnover and dynamics by several molecular activities (see numbers in figure): (1) Continued synaptic receptors (e.g., AMPA receptor) and channels activation during memory maintenance in the memory spines to regulate molecules that control actin dynamics and branching. (2) Stable actin polymerization and depolymerization ratio by actin regulatory proteins. (3) Capping proteins contribute to reduction in actin dynamics. In addition, a steady balance between capping and Arp2/3 proteins concentrations preserves a steady state structure of the branched network by controlling the number of branch points. (4) The balanced activation of actin regulatory proteins is kept steady by the receptors activation-mediated stabilization of actin regulatory proteins activity and levels in spines. The level of the proteins can be controlled by regulating trafficking and *in situ* proteins synthesis.

It is possible that the altered actin cytoskeleton that supports the newly shaped spines after learning serves as a blueprint where the newly actin monomers and nucleation proteins replenish the network continuously keeping the general structure intact based on the initial (post-learning) structure. In that manner the rapid turnover of F-actin does not affect the general structure of actin filaments in spine and therefore spine structure remains stable. Mature spines consist of a mixture of branched and linear actin filaments in their base, neck, and head that determines their structure. Thus, the length and network structure of the actin filaments should be in a steady state to maintain spines morphology. In this model (Figure 1) we suggest several conditions that can contribute to the maintenance of the actin network structure and consequently to preserving the stability of spine morphology: (1) Actin filaments length stays intact after learning by balancing the rate of actin polymerization and depolymerization. This could be achieved by controlling the activity of actin polymerization (e.g., formins) and depolymerization (e.g., cofilin) regulatory proteins as well as of capping proteins. (2) The branching of actin by Arp2/3, localized in spines (Kim et al., 2006, 2013; Rácz and Weinberg, 2008; Wegner et al., 2008; Hotulainen et al., 2009), is on these preexisting F-actins (F-actin in condition #1) keeping the actin network structure. This requires that Arp2/3 will recognize the targeted filament to be branched and that Wasp will be activated during the maintenance phase so that Arp2/3 can be assembled. (3) Alternatively, the structure of the actin network may be maintained en masse by keeping the concentration of actin regulatory proteins in stable balance. For example, the structure of the actin network is determined by the ratio between capping proteins and Arp2/3. Increasing the concentration of the capping proteins leads to an increase in the Arp2/3 mediated branching of the actin cytoskeleton network (Akin and Mullins, 2008). Such a balance may be achieved by a molecular mechanism that keeps the concentrations of the various actin regulatory proteins in spines constant. Keeping a stable concentration of a protein in spines could be achieved by: (1) *In situ* constant synthesis or suppression of synthesis of specific proteins in spines. The protein synthesis machinery exists in spines (e.g., Pierce et al., 2000; Ostroff et al., 2002) and active synapses may attract mRNAs (Kosik, 2016). Active suppression of dendritic protein synthesis is involved in miniature synaptic transmission induced stabilization of synaptic function (Sutton et al., 2006). (2) Actively controlling the translocation of specific proteins into specific spines. For example, myosin can deliver proteins into spines and may distribute distinct cargoes by using specific receptors (Kneussel and Wagner, 2013). Moreover, it has been shown that proteins can be translocated into specific spines after synaptic activation or learning (Matsuo et al., 2008; Bosch et al., 2014). Thus, learning induced continuous activation of specific spines (see above) can contribute to delivery of specific proteins and mRNAs into these spines keeping the concentrations of actin regulatory proteins in spines at steady state and thus preserving the actin cytoskeleton structure. Indeed, altering the concentration or activity of actin regulatory proteins can affect spines stability. For example, the stability of mature dendritic spines is controlled by cofilin activity and affecting this activity disrupts spines stability (e.g., Shi et al., 2009). Deletion of ArpC3 leads to a loss of large mushroom-shaped spines and an increase in filopodial-like spines indicating that conserved level of Arp2/3 may be crucial for long-term stabilization of spines *in vivo* (Kim et al., 2013).

Spines may also shrink after stimulation leading to long-term depression (LTD) that may be involved in experience-based neuronal network refinement (Nägerl et al., 2004; Zhou et al., 2004). LTD induced shrinkage of spines may lead to a new steady state with less complexed branched F-actin network and less F-actin (Okamoto et al., 2004). This may also be accompanied by reduction of AMPA receptors in synapse (Shepherd and Huganir, 2007). The stabilization of thinner spines may still be dependent on glutamate receptors or/and other synaptic receptors activities such as Eph receptors (e.g., Shi et al., 2009) balancing the new actin dynamics and branching by maintaining the concentrations and activities of actin regulatory proteins in the spine. Since there might be a decrease in calcium influx in response to synaptic stimulation, stabilizing the structure of actin cytoskeleton network may be dependent on maintaining actin regulatory proteins concentrations and activities using other signaling pathways.

## Summary and conclusions

Long-term memories last for years. The neuronal processes that encode memories last as long as memory exists. Alterations in neuronal morphology especially of dendritic spines have been suggested to underlie the formation of memory and their stabilization the maintenance of memory. In this review we show evidence indicating that changes in actin cytoskeleton subserves spine morphogenesis induced by learning and that preserving these actin cytoskeleton alterations is involved in maintaining spine morphology and memory for a long period of time. We suggest mechanisms that include reduction in actin dynamics and the formation of a stable blue print of actin cytoskeleton structure in spines to preserve actin cytoskeleton scaffold, spine morphology and memory.

## Author contributions

All authors listed have made a substantial, direct and intellectual contribution to the work, and approved it for publication.

### Conflict of interest statement

The authors declare that the research was conducted in the absence of any commercial or financial relationships that could be construed as a potential conflict of interest.
